# Plasma membrane expression of G protein-coupled estrogen receptor (GPER)/G protein-coupled receptor 30 (GPR30) is associated with worse outcome in metachronous contralateral breast cancer

**DOI:** 10.1371/journal.pone.0231786

**Published:** 2020-04-17

**Authors:** Julia Tutzauer, Martin Sjöström, Pär-Ola Bendahl, Lisa Rydén, Mårten Fernö, L. M. Fredrik Leeb-Lundberg, Sara Alkner

**Affiliations:** 1 Department of Experimental Medical Science, Lund University, Lund, Sweden; 2 Department of Clinical Sciences Lund, Division of Oncology and Pathology, Lund University, Lund, Sweden; 3 Department of Clinical Sciences Lund, Division of Surgery, Lund University, Lund, Sweden; 4 Department of Surgery, Skåne University Hospital, Lund, Sweden; 5 Department of Oncology, Skåne University Hospital, Lund, Sweden; Turun Yliopisto, FINLAND

## Abstract

**Background:**

G protein-coupled estrogen receptor (GPER), or G protein-coupled receptor 30 (GPR30), is reported to mediate non-genomic estrogen signaling. GPR30 associates with breast cancer (BC) outcome and may contribute to tamoxifen resistance. We investigated the expression and prognostic significance of GPR30 in metachronous contralateral breast cancer (CBC) as a model of tamoxifen resistance.

**Methods:**

Total GPR30 expression (GPR30_TOT_) and plasma membrane-localized GPR30 expression (GPR30_PM_) were analyzed by immunohistochemistry in primary (BC1; n_BC1_ = 559) and contralateral BC (BC2; n_BC2_ = 595), and in lymph node metastases (LGL; n_LGL1_ = 213; n_LGL2_ = 196). Death from BC (BCD), including BC death or death after documented distant metastasis, was used as primary end-point.

**Results:**

GPR30_PM_ in BC2 and LGL2 were associated with increased risk of BCD (HR_BC2_ = 1.7, *p* = 0.03; HR_LGL2_ = 2.0; *p* = 0.02). In BC1 and BC2, GPR30_PM_ associated with estrogen receptor (ER)-negativity (*p*_BC1_<0.0001; *p*_BC2_<0.0001) and progesterone receptor (PR)-negativity (*p*_BC1_ = 0.0007; *p*_BC2_<0.0001). The highest GPR30_TOT_ and GPR30_PM_ were observed in triple-negative BC. GPR30_PM_ associated with high Ki67 staining in BC1 (*p*<0.0001) and BC2 (*p*<0.0001). GPR30_TOT_ in BC2 did not associate with tamoxifen treatment for BC1. However, BC2 that were diagnosed during tamoxifen treatment were more likely to express GPR30_PM_ than BC2 diagnosed after treatment completion (*p* = 0.01). Furthermore, a trend was observed that patients with GPR30_PM_ in an ER-positive BC2 had greater benefit from tamoxifen treatment.

**Conclusion:**

PM-localized GPR30 staining is associated with increased risk of BC death when expressed in BC2 and LGL2. Additionally, PM-localized GPR30 correlates with prognostic markers of worse outcome, such as high Ki67 and a triple-negative subtype. Therefore, PM-localized GPR30 may be an interesting new target for therapeutic exploitation. We found no clear evidence that total GPR30 expression is affected by tamoxifen exposure during development of metachronous CBC, or that GPR30 contributes to tamoxifen resistance.

## Introduction

Metachronous contralateral breast cancer (CBC) is a second, presumably independent primary tumor (BC2) developed in the contralateral breast after the first breast cancer (BC1). The lifetime risk of a breast cancer (BC) patient developing CBC has been estimated at 2–20%, depending on factors such as family history, prior endocrine treatment, and age at BC1 diagnosis [1–3]. Similar to BC in general, CBC is a heterogenous disease and both disease stage and the molecular characteristics of the tumor is used to assess prognosis and benefit of therapy, where axillary lymph node (LGL) involvement is one of the strongest prognostic factors [4]. At the molecular level, the tumor is generally characterized by the expression of estrogen receptor α (ER), progesterone receptor (PR), and human epidermal growth factor receptor 2 (HER2), as well as the proliferation rate [5]. About 80% of all BC exhibit overexpression of ER, through which the female steroid hormone estrogen acts to stimulate cell growth and proliferation. Therefore, endocrine therapies directed to disrupt ER signaling are central in current BC treatment, acting either by suppressing ER activity, e.g. selective ER modulators or downregulators, or by inhibiting estrogen production, e.g. aromatase inhibitors. The selective ER modulator tamoxifen is one of the most widely prescribed endocrine agents for treatment of ER-positive BC [6]. In the adjuvant setting, 5 years of tamoxifen treatment reduces the 10-year risk of recurrence by almost 50%, and the annual risk of BC mortality by almost one-third [7, 8]. However, not all ER-positive tumors respond to tamoxifen therapy, and resistance may occur *de novo* or during treatment. Tamoxifen reduces the incidence of CBC, but CBC evolving during tamoxifen treatment is assumed to have intrinsic resistance. Efforts aimed to further understand resistance mechanisms have led to a number of important discoveries, including pathological epigenetic changes or mutations in the *ESR1* gene, and interference with other growth stimulatory signaling pathways. These mechanisms subsequently result in augmented receptor activity, ligand-independent growth and transcription, or reduced drug sensitivity [6, 9, 10]. Despite these discoveries, ER remains the only predictive marker for endocrine treatment.

G protein-coupled estrogen receptor (GPER), originally named G protein-coupled receptor 30 (GPR30), is a receptor involved in rapid, non-genomic responses to estrogen [11]. In contrast to the classical ER, which is a soluble receptor residing in the cytoplasm or cell nucleus, GPR30 is a transmembrane receptor reported to be expressed both in the plasma membrane (PM) [12] and in the endoplasmatic reticulum [13]. As an estrogen receptor, GPR30 has caught significant attention in BC research, and the relationship between GPR30 and BC outcome has been addressed in multiple studies. However, results are inconsistent, with the receptor conveying either better [14, 15] or worse prognosis [16, 17], or lacking any prognostic value [18] for BC outcome. Additionally, *in vitro* studies have shown that GPR30 is pro-apoptotic in the ER-positive BC cell line MCF-7, but proliferative in the ER-negative cell line SkBr3 [19]. Thus, GPR30 may function differently depending on the environment in which it is expressed. Both clinical and pre-clinical studies have shown that subcellular localization is also a factor influencing GPR30 function. Indeed, GPR30 staining specifically located in the PM was found to be a strong prognostic factor for poor prognosis in BC, while the total level of GPR30 staining was not [17]. Consistent with this clinical observation, an *in vitro* study showed that PM localization of GPR30 is important for receptor stimulation of ERK1/2 activity [20], a cellular signal involved in proliferation and survival. Thus, the biological context of the tumor appears to be critical for GPR30 function in BC, with subcellular localization being a factor of potential importance.

Studies have reported that GPR30 may contribute to tamoxifen resistance [21–24]. Some *in vitro* data suggest that tamoxifen directly stimulates cell growth via GPR30 [22]. This situation would be of major clinical concern, as many ER-positive BC also express GPR30, and tamoxifen treatment of these BC partly could yield increased cell growth. On the other hand, these observations may also argue that GPR30 is a potential marker to identify BC with poor responsiveness to tamoxifen. GPR30 has been suggested to function as a resistance mechanism to escape tamoxifen responsiveness. However, whether GPR30 expression changes in tumors developing resistance to tamoxifen treatment, has not yet been addressed in a larger cohort of patients.

The aim of this study was to further understand how GPR30 expression relates to BC progression, patient outcome, and previous tamoxifen treatment. To this end, we used a unique retrospective cohort of patients with CBC, either naïve or exposed to tamoxifen following BC1, with paired expression data from primary tumors and lymph node metastases, as a stepwise model of tamoxifen resistance.

## Materials and methods

### Patient cohort and TMA preparation

**Fig 1:**
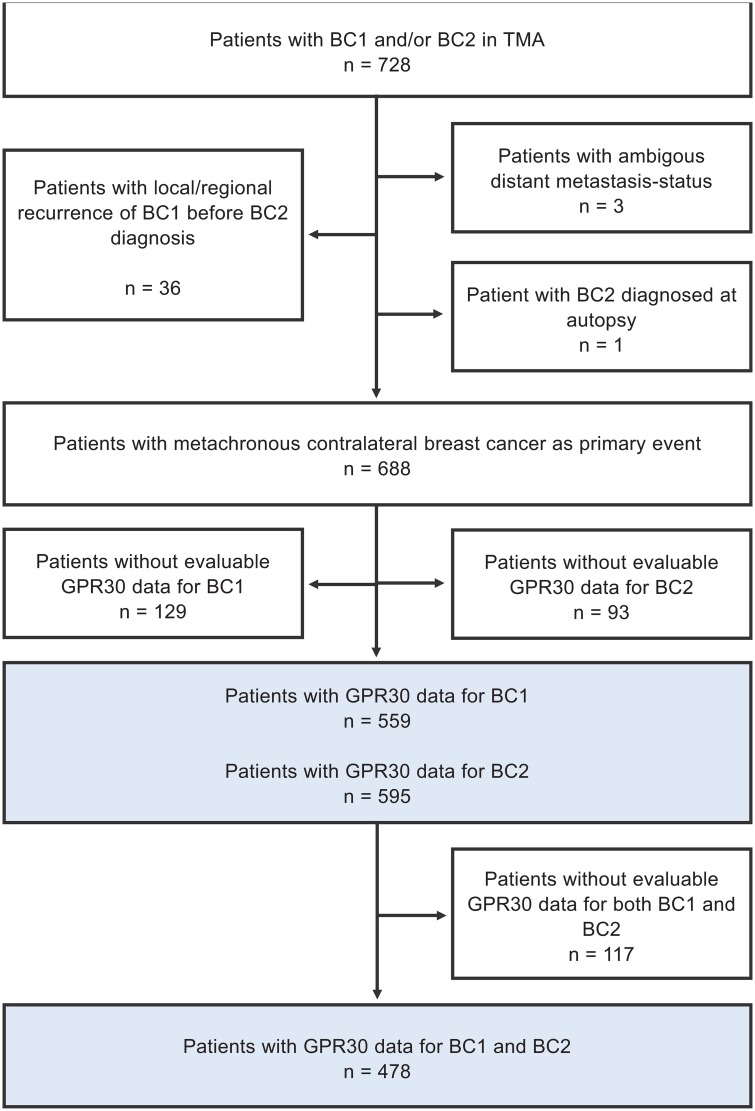
Flow-chart of inclusion and exclusion for the study cohort.

A previously constructed tissue-microarray (TMA) from 728 patients diagnosed with CBC between 1977 and 2007 at 14 hospitals within the Southern Swedish Healthcare Region was used. Details of TMA construction have been previously described [25]. Patient inclusion and number of CBC successfully stained and scored for GPR30 are summarized in Fig 1. Follow-up information was retrieved from patient charts, and cause of death and overall survival data were accessed from the Swedish National Board of Health and Welfare in March 2014. Evaluation of ER, PR, Ki67 and HER2 have been previously described [26, 27].

### Cell construction and culture

HFF11 cells, originally constructed from HeLa cells (American Type Culture Collection, ATCC), were kindly provided by K. Kotarsky [28]. HFF11 cells stably expressing the T-Rex system (HeLa TET-On/Off cells), in which the expression of human GPR30 is under the strict control by tetracycline, were constructed according to vendor instructions (ThermoFisher Scientific). MCF7 cells were obtained from ATCC. Cells were confirmed to be mycoplasma-free by the MycoSEQ^™^ Mycoplasma Detection System (ThermoFisher Scientific). HeLa TET-On/Off cells were grown in Dulbecco’s modified Eagle’s medium (DMEM; Invitrogen, Carlsbad, CA), 10% fetal calf serum (FCS; HyClone Laboratories, Logan, UT), and 1% penicillin/streptomycin (Sigma-Aldrich), using blastidine and zeocin as clone selection markers. MCF7 cells were grown in DMEM supplemented with glucose and pyruvate, and with 10% FCS. Both cells were grown in 5% CO_2_ at 37°C.

### GPR30 antibody validation

The specificity of the polyclonal goat GPR30 antibody (AF 5534; R&D Systems) was analyzed in MCF7 human breast cancer cells (American Type Culture Collection), a cell line used extensively to study native GPR30 [19]. Immunoblotting showed that the antibody recognized a receptor species with a molecular mass of 50–55 kDa in MCF7 cells (S1 Fig, panel A), consistent with that described by the vendor (R&D Systems). To ensure that the antibody reactivity was absolutely dependent on GPR30 expression, we immunoblotted HeLa TET-On/Off cells treated with and without 0.1 μM tetracycline. In these cells, the GPR30 antibody recognized three broad receptor species at about 50 kDa, 80 kDa, and 130 kDa following tetracycline treatment, whereas no significant immunoreactivity was observed in the absence of tetracycline treatment (S1 Fig, panel B). These results, and those published by us previously [15, 29], show that GPR30 antibody immunoreactivity is completely dependent on GPR30 expression. Slightly different molecular masses of the recognized receptor species were observed in native MCF7 cells and recombinant HeLa cells. This is common among G protein-coupled receptors (GPCR) and due to variations in posttranslational modifications (e.g. glycosylation, oligomerization, etc.). Confocal immunofluorescence microscopy of MCF7 cells stained live with the GPR30 antibody showed that the antibody detected receptors in the PM in these cells (S1 Fig, panel C). A similar subcellular localization was revealed using M1 FLAG antibodies (Sigma-Aldrich) to detect recombinant human GPR30 tagged at the N terminus with the FLAG epitope transiently expressed in MCF7 cells (S1 Fig, panel D). Thus, the GPR30 antibody is capable of recognizing GPR30 localized in the PM.

### IHC staining and scoring of GPR30

**Fig 2:**
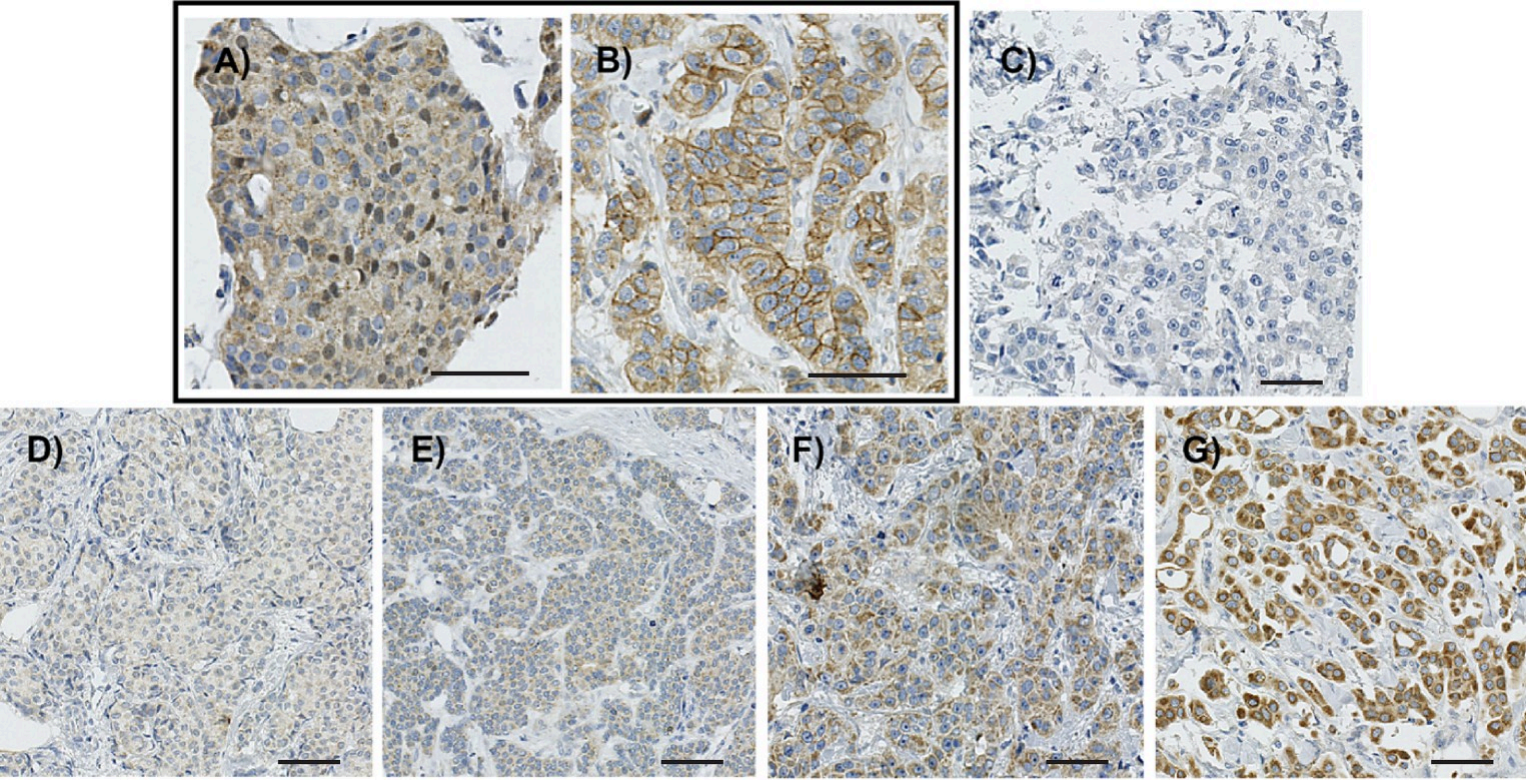
Representative images of GPR30 staining in BC samples. *A* and *B*, images of GPR30 staining intensity level 3 without plasma-membrane staining (GPR30_PM_-) (*A*) and with plasma-membrane staining (GPR30_PM_+) (*B*). *C*-*G*, images of GPR30 total staining (GPR30_TOT_) as negative (*C*), very weak (level 1) (*D*), weak (level 2) (*E*), moderate (level 3) (*F*) and very strong (level 4) (*G*). *Bar*, 50 μm.

GPR30 expression was monitored by immunohistochemical (IHC) staining with GPR30 antibody (1:50) on 1.0 mm TMA cores. Receptor expression was evaluated as total cellular staining intensity (GPR30_TOT;_ Fig 2A) and PM-specific staining intensity (GPR30_PM_; Fig 2B). PM-specific staining was defined as a clear increase of immunoreactivity on cell borders, as compared to the cytoplasm. GPR30_TOT_ was scored as overall intensity at 5 levels (Fig 2C–2G; 0 = negative; 1 = very weak; 2 = weak; 3 = moderate; 4 = strong), and GPR30_PM_ at 3 levels (0 = no increase as compared to the cytoplasmic staining; 1 = weak PM-specific staining; and 2 = strong PM-specific staining). Percentage of stained tumor cells was scored, but as the vast majority of tumors had either 0% or >50% stained cells, the intensity was used for further analysis. The staining was visually evaluated by two independent investigators (M.S., K.L.), a well-established widely accepted method to evaluate IHC staining on TMA cores, and the mean score was used, rounding to nearest integer. Due to group sizes, GPR30_TOT_ intensity was combined to two groups of weak (levels 0–2) *vs*. strong (levels 3–4). GPR30_PM_+ scores were combined to create a binary variable (levels 0 *vs*. 1–2).

### Statistical analysis

Death from BC (BCD), including BC death or death after documented distant metastasis, was used as primary end-point. Median follow-up time for patients alive at last follow-up was 9.1 years from BC2 diagnosis. Statistical analyses were carried out in RStudio 1.1.442 (RStudio, Boston, MA, USA) using R 3.5.1 [30]. Associations between GPR30 and factors such as patient attributes and clinicopathological markers were evaluated using Pearson’s χ^2^-test or Mantel-Haenszel χ^2^-test for trend. GPR30 staining in paired tumors was compared using Wilcoxon matched pairs signed rank test. Cumulative incidence of BCD was calculated with death from other causes as competing risk event using the cmprsk R package [31]. Cox proportional hazards models with Wald test was used to calculate hazard ratios (HR), using the survival R package [32]. Proportional hazards were assessed using Schoenfeldt residuals. As the assumption was not reasonably well met in all the analyses, HRs should be cautiously interpreted as average effects over the follow-up interval, which was restricted to maximum 10 years to reduce the problem of non-proportional hazards.

### Ethical approval

All procedures performed in studies involving human participants were approved by the Regional Ethical Review Board of Lund University (LU240-01) and in accordance with the 1964 Helsinki declaration and its later amendments or comparable ethical standards.

### Informed consent

Since the study handled saved paraffin material, often several decades old, informed consent was not possible to retrieve from all patients. Nevertheless, all data was analyzed and presented anonymously, and a note was published in the local paper, informing previous BC patients to contact the research group if they did not want their tumor tissue to be used in scientific studies. This procedure was accepted by the Regional Ethical Review Board of Lund University (LU240-01).

## Results

### GPR30 expression in relation to patient and tumor characteristics

**Fig 3:**
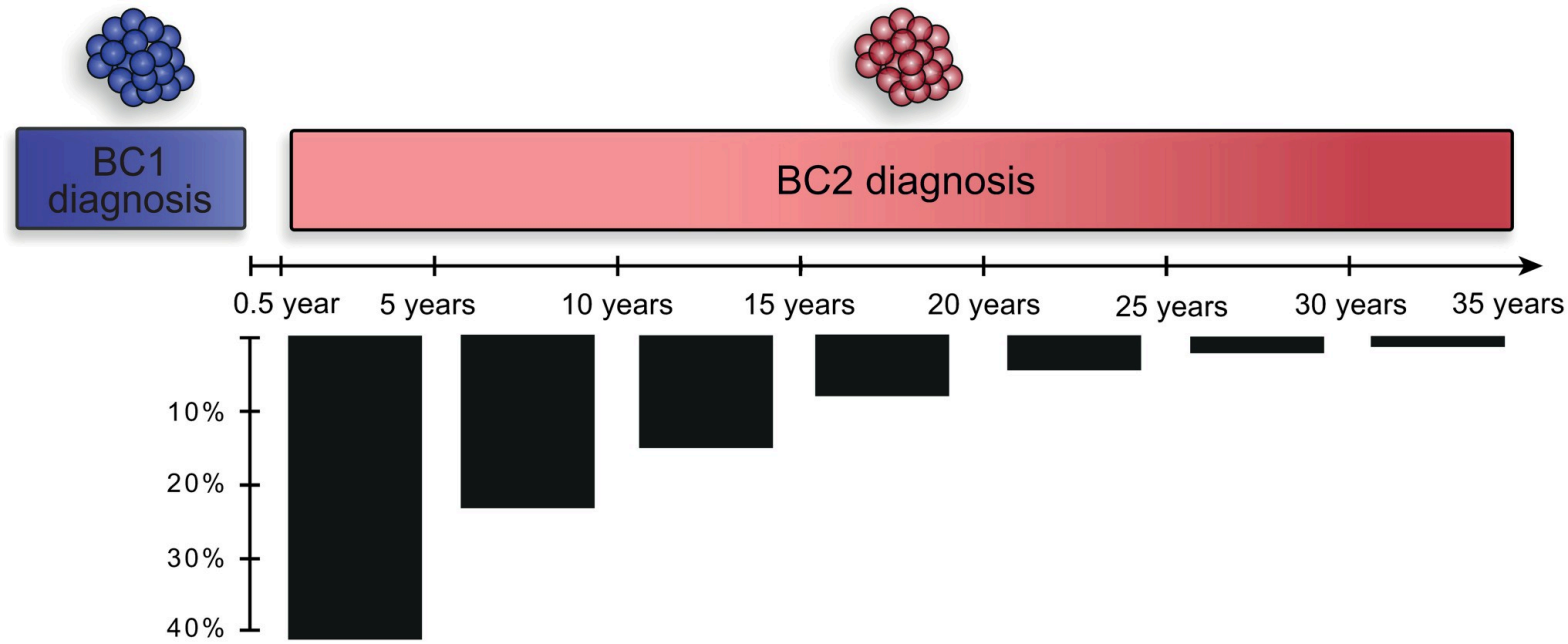
Timeline of CBC in the study cohort. Timeline showing the distribution of the interval between BC1 and BC2 in the study cohort.

GPR30 expression was assessed in 688 women with metachronous CBC, where BC2 was diagnosed between 6 months and 34.1 years after BC1 diagnosis (Fig 3, median = 6.6 years). Receptor expression was monitored by immunohistochemistry in two variables; receptor staining at the PM (GPR30_PM_+; Fig 2A and 2B) and total receptor staining intensity (GPR30_TOT_; Fig 2C–2G). Increasing GPR30_TOT_ was associated with a higher fraction of GPR30_PM_+ tumors in both BC1 and BC2 (Table 1). As previously observed in three other cohorts [17], GPR30_TOT_ associated with ER and PR expression in a biphasic manner in both BC1 and BC2, where tumors with no or very weak GPR30_TOT_ and tumors with strong GPR30_TOT_ were more likely to be ER-negative and PR-negative, whereas tumors with weak or moderate GPR30_TOT_ were more likely to be ER-positive and PR-positive (Table 1). Interestingly, GPR30_PM_+ staining associated strongly with ER-negative and PR-negative status in both BC1 and BC2. GPR30_PM_+ status also strongly associated with high Ki67 staining in both BC1 and BC2 in this cohort. Lastly, both GPR30_TOT_ and GPR30_PM_ correlated with tumor subtype, with strong GPR30_TOT_ and GPR30_PM_+ status being significantly more prevalent among triple-negative cancers in both BC1 and BC2 (Table 1).

**Table 1:** Association between total GPR30 intensity score (GPR30_TOT_), plasma membrane GPR30 (GPR30_PM_) status, and various clinicopathological variables.

**BC1** n = 559	**GPR30**_**TOT**_ **intensity of BC1; n (%)**						**GPR30**_**PM**_**+ BC1; n (%)**			
	0	1	2	3	4	*p*	No PM GPR30 505 (90)	PM+ GPR30 54 (10)	*p*	Missing GPR30 data
	46 (8)	141 (25)	263 (47)	103 (18)	6 (1)					
**BC1 diagnosis**										
<1977 (n = 122)	11 (13)	24 (28)	36 (42)	14 (17)	0 (0)	0.703	74 (87)	11 (13)	0.52	37
1977–1986 (n = 210)	14 (8)	45 (27)	79 (47)	26 (16)	3 (2)		149 (89)	18 (11)		43
1987–1996 (n = 265)	15 (7)	55 (25)	108 (48)	43 (19)	2 (<1)	0.020^a^	204 (92)	19 (8)		42
1997–2007 (n = 91)	6 (7)	17 (20)	40 (48)	20 (24)	1 (1)		78 (93)	6 (7)		7
**Age at BC1 diagnosis**										
<50 years (n = 189)	18 (12)	43 (30)	63 (43)	19 (13)	3 (2)	0.027	127 (87)	19 (13)	0.15	43
≥50 years (n = 499)	28 (7)	98 (24)	200 (48)	84 (20)	3 (<1)	0.011^a^	378 (92)	35 (8)		86
**Interval between tumors**										
<5 years (n = 293)	21 (8)	61 (24)	115 (46)	51 (20)	4 (2)	0.67	222 (88)	30 (12)	0.14	41
≥5 years (n = 395)	25 (8)	80 (26)	148 (48)	52 (17)	2 (<1)	0.37^a^	283 (92)	24 (8)		88
**Histological type BC1**										
Ductal (n = 391)	21 (6)	83 (25)	167 (50)	58 (17)	4 (1)	0.26	298 (90)	35 (10)	0.01	58
Lobular (n = 95)	11 (14)	20 (25)	31 (38)	19 (24)	0 (0)	0.22^a^	79 (98)	2 (2)		14
Other (n = 125)	9 (9)	25 (26)	46 (48)	16 (17)	0 (0)		93 (97)	3 (3)		29
Missing (n = 77)	5	13	19	10	2		35	14		28
**Tumor subclass**										
Luminal A like (n = 401)	25 (7)	95 (25)	193 (51)	63 (17)	0 (0)	<0.0001	354 (94)	22 (6)	<0.0001	25
Luminal B like (n = 69)	2 (3)	15 (22)	36 (52)	16 (23)	0 (0)	0.93^a^	63 (91)	6 (9)		0
HER2+ luminal (n = 17)	1 (6)	2 (12)	10 (59)	4 (24)	0 (0)		13 (77)	4 (24)		0
HER2+ non-luminal (n = 13)	1 (8)	3 (23)	8 (62)	1 (8)	0 (0)		11 (85)	2 (15)		0
Triple-negative (n = 63)	12 (20)	19 (31)	11 (18)	13 (21)	6 (10)		43 (71)	18 (29)		2
Missing (n = 125)	5	7	5	6	0		21	2		102
**Node status**										
N0 (n = 416)	23 (7)	88 (26)	157 (46)	69 (20)	4 (1)	0.28	305 (89)	36 (11)	0.35	75
N+ (n = 213)	17 (9)	47 (26)	94 (51)	25 (14)	1 (<1)	0.11^a^	170 (92)	14 (8)		29
Missing (n = 59)	6	6	12	9	1		30	4		25
**Size**										
≤20 mm (n = 404)	19 (6)	79 (24)	160 (48)	68 (21)	5 (1)	0.047	298 (90)	33 (10)	0.76	73
>20 mm (n = 215)	23 (12)	54 (28)	86 (44)	30 (16)	1 (<1)	0.0033^a^	177 (91)	17 (9)		21
Missing (n = 69)	4	8	17	5	0		29	5		35
**ER status**										
<10% stained (n = 99)	17 (18)	29 (31)	23 (25)	19 (20)	6 (6)	<0.0001	73 (78)	21 (22)	<0.0001	5
≥10% stained (n = 494)	28 (6)	112 (24)	240 (52)	83 (18)	0 (0)	0.11^a^	431 (93)	32 (7)		31
Missing (n = 95)	1	0	0	1	0		1	1		93
**PR status**										
<10% stained (n = 165)	22 (14)	41 (26)	56 (36)	32 (20)	6 (4)	<0.0001	131 (83)	26 (17)	0.00070	8
≥10% stained (n = 428)	23 (6)	100 (25)	207 (52)	70 (18)	0 (0)	0.38^a^	373 (93)	27 (7)		28
Missing (n = 95)	1	0	0	1	0		1	1		93
**HER2 staining**										
Negative (n = 544)	42 (8)	133 (26)	241 (47)	93 (18)	6 (1)	0.86	469 (91)	46 (9)	0.13	29
Positive (n = 40)	3 (7)	8 (20)	21 (53)	8 (20)	0 (0)	0.63^a^	33 (83)	7 (17)		0
Missing (n = 104)	1	0	1	2	0		3	1		100
**Ki67 staining**										
<20% stained (n = 356)	30 (9)	85 (26)	160 (48)	57 (17)	1 (<1)	0.19	315 (95)	18 (5)	<0.0001	23
≥20% stained (n = 227)	15 (7)	56 (25)	102 (46)	44 (20)	5 (2)	0.013^a^	187 (84)	35 (16)		5
Missing (n = 105)	1	0	1	2	0		3	1		101
**PM GPR30 status**										
Negative (n = 505)	46 (9)	137 (27)	235 (47)	87 (17)	0 (0)	<0.0001	-	-	-	0
Positive (n = 54)	0 (0)	4 (7)	28 (52)	16 (30)	6 (11)	<0.0001^a^	-	-		0
**BC2** n = 595	**GPR30**_**TOT**_ **intensity of BC2; n (%)**						**GPR30**_**PM**_**+ BC2; n (%)**			
	0	1	2	3	4	*p*	No PM GPR30 557 (94)	PM+ GPR30 38 (6)	*p*	Missing GPR30data
	31 (5)	151 (25)	285 (48)	117 (20)	11 (2)					
**CBC diagnosis**										
1977–1986 (n = 136)	6 (6)	27 (25)	45 (42)	28 (26)	1 (<1)	0.072	96 (90)	11 (10)	0.18	29
1987–1996 (n = 243)	11 (5)	66 (32)	89 (44)	33 (16)	5 (3)	0.48^a^	192 (94)	12 (6)	0.10^1^	39
1997–2007 (n = 309)	14 (5)	58 (20)	151 (53)	56 (20)	5 (2)		269 (95)	15 (5)		25
**Age at CBC diagnosis**										
<50 years (n = 67)	1 (2)	18 (31)	26 (45)	12 (21)	1 (2)	0.65	53 (91)	5 (9)	0.65	9
≥50 years (n = 621)	30 (6)	133 (25)	259 (48)	105 (20)	10 (2)	0.84^a^	504 (94)	33 (6)		84
**Interval between tumors**										
<5 years (n = 293)	11 (5)	64 (26)	118 (48)	50 (20)	4 (2)	0.95	230 (93)	17 (7)	0.81	46
≥5 years (n = 395)	20 (6)	87 (25)	167 (48)	67 (19)	7 (2)	0.79^a^	327 (94)	21 (6)		47
**Histology BC2**										
Ductal (n = 450)	24 (6)	108 (27)	189 (46)	82 (20)	5 (1)	0.022	377 (92)	31 (8)	0.031	42
Lobular (n = 130)	2 (2)	32 (29)	59 (53)	17 (15)	2 (2)	0.032^a^	111 (99)	1 (1)		130
Other (n = 77)	2 (4)	8 (15)	28 (52)	12 (22)	4 (7)		51 (94)	3 (6)		77
Missing (n = 31)	3	3	9	6	0		18	3		10
**Tumor subclass**										
Luminal A like (n = 403)	16 (4)	88 (23)	193 (51)	75 (20)	4 (1)	<0.0001	367 (98)	9 (2)	<0.0001	27
Luminal B like (n = 88)	4 (5)	25 (28)	48 (55)	11 (13)	0 (0)	0.76^a^	83 (94)	5 (6)		0
HER2+ luminal (n = 19)	0 (0)	5 (26)	10 (53)	4 (21)	0 (0)		17 (90)	2 (10)		0
HER2+ non-luminal (n = 10)	0 (0)	3 (30)	3 (30)	4 (40)	0 (0)		9 (90)	1 (10)		0
Triple-negative (n = 74)	7 (10)	19 (27)	19 (27)	18 (26)	7 (10)		50 (71)	20 (29)		4
Missing (n = 94)	4	11	12	5	0		31	1		94
**Node status**										
N0 (n = 371)	15 (5)	80 (26)	155 (50)	58 (19)	5 (2)	0.39	290 (93)	23 (7)	0.54	58
N+ (n = 196)	13 (7)	54 (30)	74 (41)	38 (21)	3 (2)	0.43^a^	172 (95)	10 (5)		14
Missing (n = 121)	3	17	56	21	3		95	5		121
**Size**										
≤20 mm (n = 481)	19 (5)	96 (23)	199 (48)	91 (22)	7 (2)	0.13	387 (94)	25 (6)	0.61	69
>20 mm (n = 181)	11 (7)	53 (31)	76 (45)	26 (15)	4 (2)	0.027^a^	157 (92)	13 (8)		11
Missing (n = 13)	1	2	10	0	0		13	0		13
**ER status**										
<10% stained (n = 105)	8 (8)	30 (30)	28 (28)	26 (26)	7 (7)	<0.0001	77 (78)	22 (22)	<0.0001	6
≥10% stained (n = 524)	21 (4)	120 (24)	256 (52)	91 (19)	4 (<1)	0.47^a^	476 (97)	16 (3)		32
Missing (n = 59)	2	1	1	0	0		4	0		55
**PR status**										
<10% stained (n = 212)	13 (7)	49 (25)	90 (45)	39 (20)	9 (5)	0.0097	175 (88)	25 (12)	<0.0001	12
≥10% stained (n = 412)	16 (4)	99 (26)	192 (50)	78 (20)	2 (<1)	0.62^a^	375 (97)	12 (3)		25
Missing (n = 8)	2	3	3	0	0		7	1		56
**HER2 status**										
Negative (n = 552)	29 (5)	139 (25)	268 (49)	105 (19)	11 (2)	0.24	517 (94)	35 (6)	0.93	32
Positive (n = 37)	0 (0)	11 (30)	15 (40)	11 (30)	0 (0)	0.37^a^	34 (92)	3 (8)		0
Missing (n = 67)	2	1	2	1	0		6	0		67
**Ki67 staining**										
<20% stained (n = 349)	18 (6)	73 (23)	153 (48)	69 (22)	6 (2)	0.40	311 (98)	8 (3)	<0.0001	30
≥20% stained (n = 266)	10 (4)	76 (29)	126 (48)	47 (18)	5 (2)	0.39^a^	234 (89)	30 (11)		2
Missing (n = 73)	3	2	6	1	0		12	0		61
**Radiotherapy BC1**										
No (n = 257)	9 (4)	52 (23)	119 (52)	43 (19)	5 (2)	0.41	218 (96)	10 (4)	0.15	29
Yes (n = 425)	22 (6)	97 (27)	163 (45)	74 (20)	6 (2)	0.28^a^	334 (92)	28 (8)		63
Missing (n = 5)	0	2	3	0	0		5	0		1
**Chemotherapy for BC1**										
No (n = 615)	27 (5)	135 (25)	253 (48)	105 (20)	10 (2)	0.98	496 (94)	34 (6)	1.0	85
Yes (n = 66)	4 (7)	14 (24)	29 (49)	11 (18)	1 (2)	0.79^a^	55 (93)	4 (7)		7
Missing (n = 7)	0	2	3	1	0		6	0		1
**Tamoxifen for BC1**										
All patients										
No tamoxifen (n = 467)	25 (5)	115 (25)	226 (48)	92 (20)	9 (2)	0.96	435 (93)	32 (7)	0.57	73
Tamoxifen (n = 122)	6 (5)	34 (28)	56 (46)	24 (20)	2 (2)	0.73^a^	116 (95)	6 (5)		19
Missing (n = 7)	0	2	3	1	0		6	0		1
* Tamoxifen treated for BC1*										
BC2 diagnosis during treatment (n = 60)	1 (2)	18 (35)	19 (37)	12 (23)	2 (4)	0.089	46 (89)	6 (11)	0.013	11
BC2 diagnosis after treatment (n = 81)	5 (7)	16 (23)	37 (53)	12 (17)	0 (0)		70 (100)	0 (0)		8
* Not tamoxifen treated for BC1*										
BC2<5 years after BC1 (n = 222)	10 (5)	45 (24)	92 (49)	39 (21)	2 (1)	0.83	177 (94)	11 (6)	0.61	34
BC2>5 years after BC1 (n = 318)	15 (5)	70 (25)	134 (48)	53 (19)	7 (3)	0.99^a^	258 (93)	21 (7)		39
**PM GPR30 status**										
Negative (n = 557)	31 (6)	149 (27)	270 (48)	103 (19)	4 (<1)	<0.0001				
Positive (n = 38)	0 (0)	2 (5)	15 (40)	14 (37)	7 (18)	<0.0001^a^				

**Abbreviations**: BC: breast cancer; BC1: the first primary BC; BC2: the second primary BC; CBC: contralateral breast cancer; GPR30: G protein-coupled receptor 30; PM: plasma membrane; HER2: human epidermal growth factor receptor-2; N+/N0: presence or absence of lymph node metastases; ER: estrogen receptor α; PR: progesterone receptor.

Values for p are calculated using Pearson’s χ^2^-test without continuity correction if otherwise is not stated.

^a^ Calculated using Mantel-Haenszel χ^2^-test test (χ^2^-test for trend).

**Fig 4:**
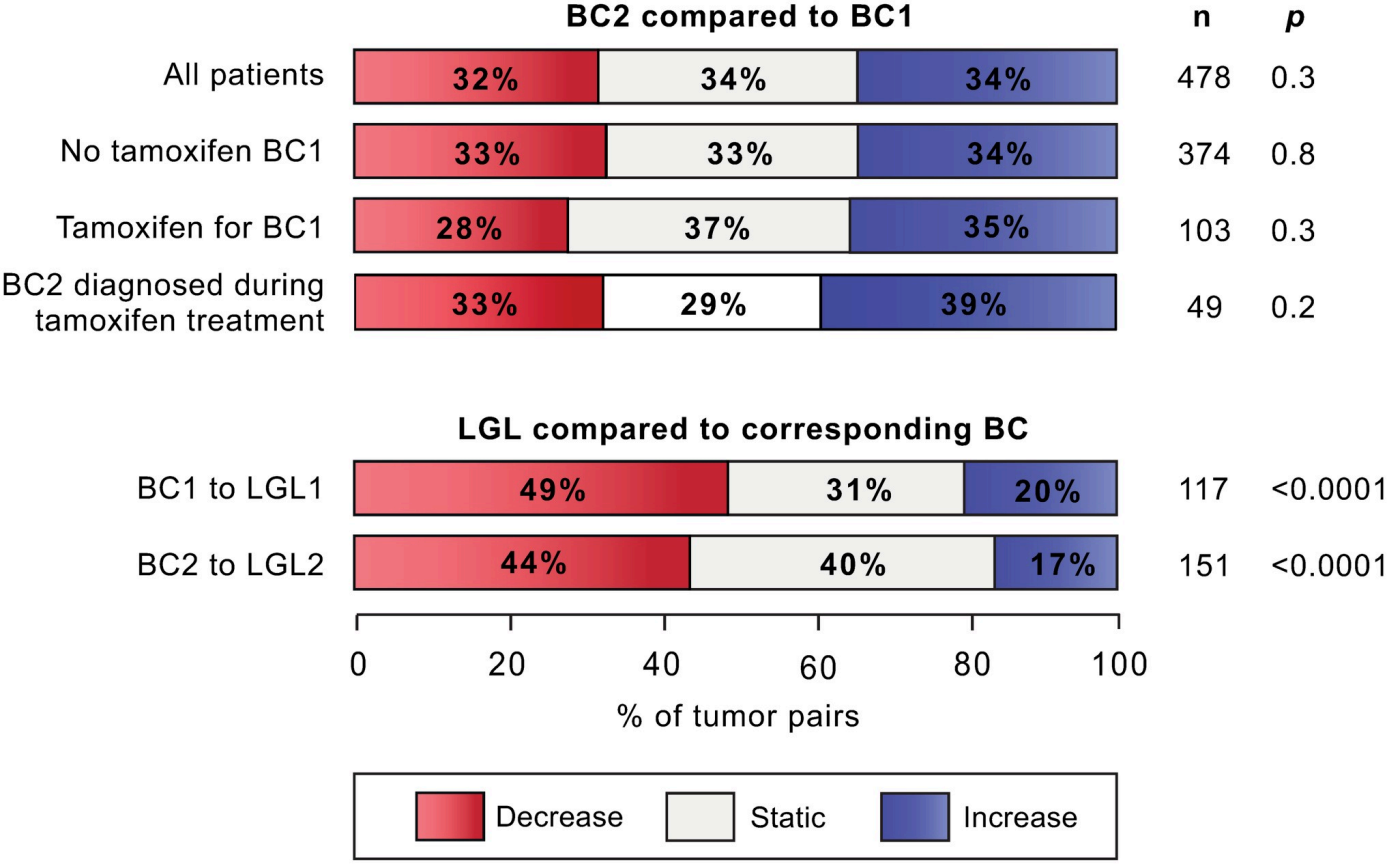
GPR30 staining in paired tumors. The change in GPR30 intensity (level 0–4) between paired BC and LGL. In relation to the tumor assumed to have developed earlier, the intensity shift in the second tumor is characterized as decreasing, stable or increasing for each tumor pair respectively. The intensity shift was assessed statistically using Wilcoxon matched pairs signed rank test.

To assess GPR30 expression through tumor progression, GPR30_TOT_ staining was compared between BC1 and BC2, and with their corresponding LGL (Fig 4). A majority of the LGLs had a weaker GPR30_TOT_ compared to the primary BC in both the BC1/LGL1 pair (Fig 4; *p*<0.0001) and the BC2/LGL2 pairs (*p*<0.0001).

### GPR30 expression and risk of BCD in CBC patients

**Fig 5:**
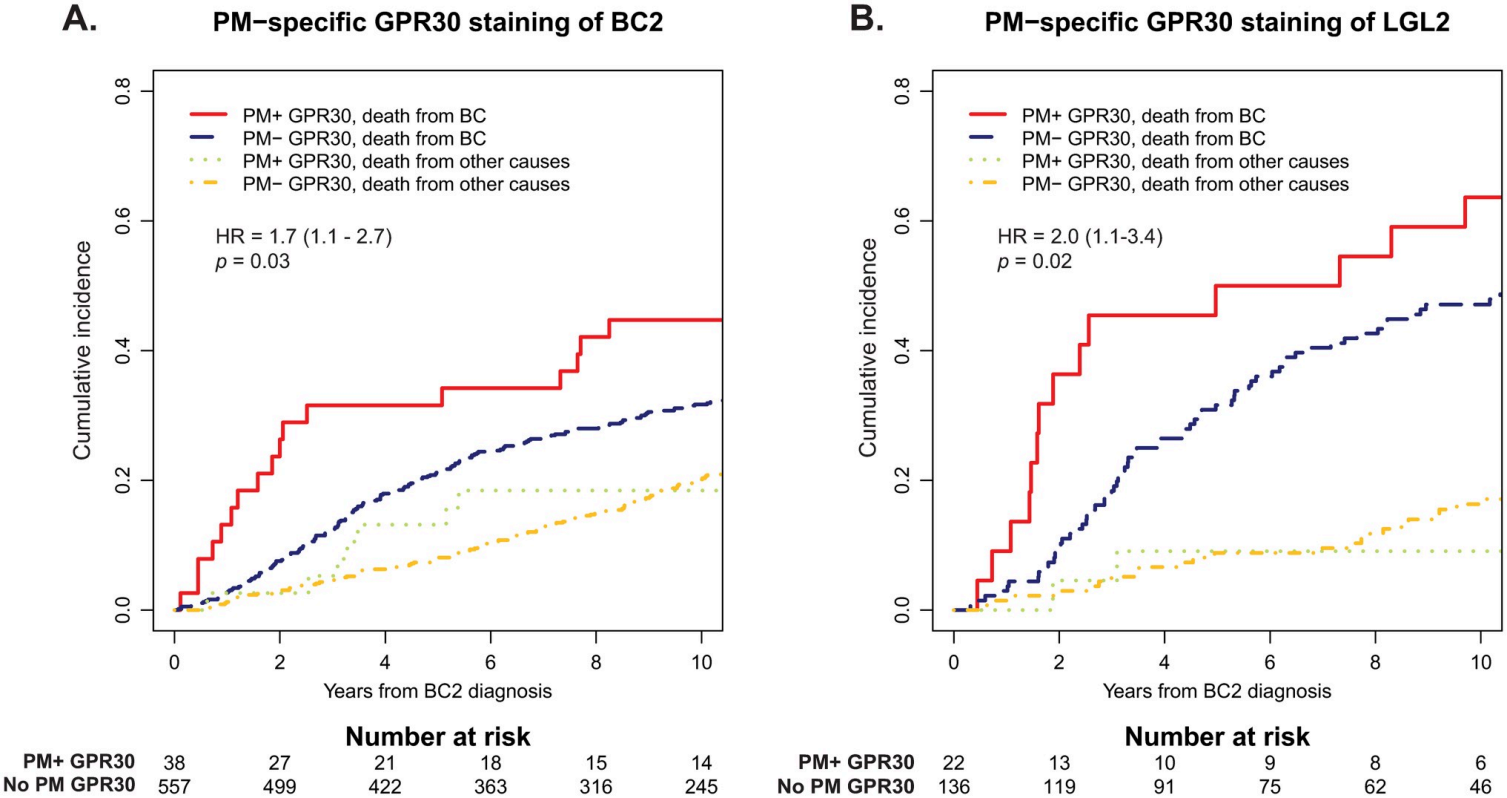
Cumulative incidence of BCD in relation to PM-specific GPR30 staining (GPR30_PM_). *A*, cumulative incidence of BCD in relation to GPR30_PM_ staining of BC2. *B*, cumulative incidence of BCD in relation to GPR30_PM_ staining of LGL2. In *A* and *B*, cumulative incidences of competing event (death from other cause than BC) are shown for comparison.

**Fig 6:**
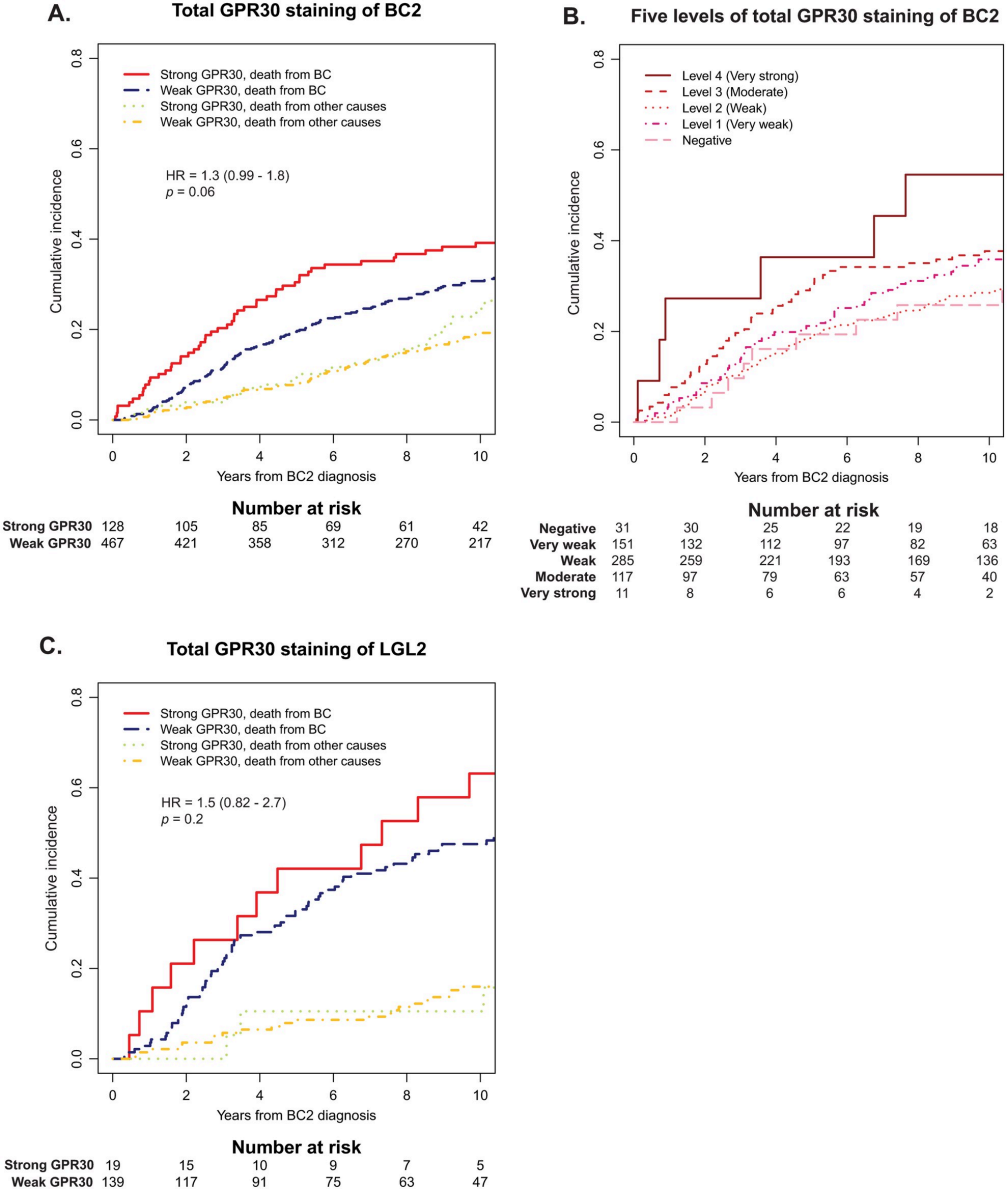
Cumulative incidence of BCD in relation to total GPR30 staining (GPR30_TOT_). *A*, cumulative incidence of BCD in relation to GPR30_TOT_ of BC2 in the whole cohort. *B*, cumulative incidence of BCD in relation to the five GPR30 staining intensity levels of BC2. *C*, cumulative incidence of BCD in relation to GPR30_TOT_ of LGL2. In *A*-*C*, cumulative incidences of competing event (death from other cause than BC) are shown for comparison.

Previous studies by our group showed that characteristics of BC2 have the highest influence on prognosis after development of CBC, although the characteristics of BC1 continue to have some prognostic impact [33]. GPR30_PM_+ staining in both BC2 and LGL2 was associated with increased risk of BCD (Table 2; Fig 5A and 5B; HR_BC2_ = 1.7; 95% CI = 1.1–2.7; *p* = 0.03, and HR_LGL2_ = 2.0; 95% CI = 1.1–3.4; *p* = 0.02). A trend was also observed that higher GPR30_TOT_ associated with increased BCD in BC2 and LGL2 (Fig 6A–6C; HR_BC2_ = 1.3; 95% CI = 0.99–1.8; *p* = 0.06, and HR_LGL2_ = 1.5; 95% CI = 0.82–2.7; *p* = 0.2, respectively). Similar trends were seen when looking at GPR30 expression in BC1.

**Table 2:** Prognostic effect of total GPR30 staining (GPR30_TOT_) and PM-specific GPR30 (GPR30_PM_) staining of BC2 and LGL2 calculated by Cox proportional hazards model with Wald test.

		Unadjusted					Adjusted for BC1^a^					Adjusted for BC1 and BC2^b^				
	Tumor	HR	n	Events	95% CI	*p*	HR	n	Events	95% CI	*p*	HR	n	Events	95% CI	*p*
**GPR30**_**TOT**_	**BC2**	1.3	595	237	0.99–1.8	0.06	1.2	428	171	0.85–1.8	0.3	1.4	349	145	0.93–2.1	0.11
	**LGL2**	1.5	151	83	0.82–2.7	0.2	1.6	119	70	0.74–3.3	0.2	2.2	110	67	1.0–4.7	0.05
**GPR30**_**PM**_**+**	**BC2**	1.7	595	237	1.1–2.7	0.03	1.5	428	171	0.87–2.7	0.1	1.6	349	145	0.89–3.0	0.1
	**LGL2**	2.0	151	83	1.1–3.4	0.02	1.4	119	70	0.67–2.8	0.4	3.0	110	67	1.43–6.1	0.004

^a^ Adjusted for the following variables in BC1: GPR30 intensity, tumor size, node status, ER status, HER2 status and ki67 staining.

^b^ Adjusted for factors in ^a^ and for the following factors in BC2: size, node status, ER status, HER2 status and Ki67 staining, plus age and calendar interval at BC2 diagnosis and interval between BC1 and BC2.

The trend that GPR30 is a negative prognostic marker for BCD remained in multivariate analysis (Table 2), although effect sizes generally decreased. When estimating the prognostic impact of GPR30 in multivariate analyses adjusted for each variable individually, the prognostic effect of GPR30 was found to mainly be affected when adjusting for ER-status (S1 Table). Interestingly, stratified survival analyses based on ER-status showed that both strong GPR30_TOT_ and GPR30_PM_+ staining in BC2 were graphically associated with increased BCD among patients with ER-positive tumors, but not ER-negative tumors (S2 Fig and S2 Table). However, statistical test for interaction did not show a significant difference in the prognostic value of GPR30 between ER-positive and ER-negative tumors (GPR30_TOT_ *p*_interaction_ = 0.7; GPR30_PM_+ *p*_interaction_ = 0.1). To address the combined prognostic information of GPR30 status in both BC1 and BC2, we created combination variables with information from paired BC1 and BC2 for GPR30_TOT_ and GPR30_PM_ respectively. This analysis showed that, although BC2 carried the higher prognostic value, BC1 also added prognostic information (S3 Fig and S3 Table).

### GPR30 expression and tamoxifen treatment

To address the GPR30 expression in individual CBC cases, we matched the GPR30 variables of BC1 and BC2 within each patient individually. We observed that GPR30_TOT_ was equally likely to have increased, decreased, or remained the same in BC2 compared to BC1 (Fig 4). Interestingly, the pattern was similar regardless if tamoxifen treatment had been given for BC1 or not (Fig 4). In the whole cohort, there was no association having received tamoxifen treatment for BC1, and score of GPR30_TOT_ or GPR30_PM_+ in BC2. However, in a subset of patients diagnosed with BC2 during adjuvant tamoxifen treatment for BC1, BC2 were more likely to be GPR30_PM_+ than in BC2 diagnosed after completed tamoxifen treatment (11% vs 0%, *p* = 0.01; Table 1).

When stratified for tamoxifen treatment of ER-positive BC2, GPR30_PM_+ exhibited higher prognostic potential in patients that did not receive tamoxifen for BC2 as compared to patients that did receive tamoxifen. The prognostic potential remained significant in both univariate analysis and multivariate analysis adjusting for attributes of BC1 and BC2 (S2 Table; univariate HR = 2.8, 95% CI = 1.3–6.0, *p* = 0.01; multivariate HR = 3.8, 95% CI = 1,4–11, *p* = 0.01). Additionally, when studying the effect of tamoxifen on ER-positive BC2, there was a trend that patients with strong GPR30_TOT_ or GPR30_PM_+ staining had a greater benefit from tamoxifen treatment than those with lower GPR30_TOT_ or without GPR30_PM_ staining (S4 Fig). However, interaction between GPR30 and tamoxifen could not be confirmed statistically (S2 Table; GPR30_TOT_ *p*_interaction_ = 0.4; GPR30_PM_+ *p*_interaction_ = 0.3). Finally, the prognostic effect of GPR30 in BC2 did not seem to be affected by whether tamoxifen had been given for BC1 or not (GPR30_TOT_ *p*_interaction_ = 0.2; GPR30_PM_+ *p*_interaction_ = 0.4).

## Discussion

GPR30 is a G protein-coupled receptor reported to mediate non-genomic estrogenic signaling and contribute to BC progression and tamoxifen resistance. However, the literature is inconsistent regarding the pathophysiological profile of GPR30 in BC, and the receptor function is still poorly understood. In this study, we sought to clarify the role of GPR30 during BC development and progression with or without tamoxifen exposure. To this end, we used a unique retrospective cohort of CBC, serving as a model of tamoxifen resistance. We show in this material that GPR30 staining is a strong prognostic factor for increased risk of BCD, particularly when expressed in the PM. We also show that the total GPR30 staining generally decreases during tumor progression. Interestingly, we find that total GPR30 staining was unrelated to tamoxifen treatment during tumor development, and we found no clear relationship between the prognostic value of GPR30 and tamoxifen treatment.

Studies have indicated that GPR30 is influenced by the ER modulator tamoxifen, and this has been suggested to contribute to tamoxifen resistance [21–24]. Upregulation of GPR30 expression was observed following tamoxifen treatment in a small BC cohort [21], and GPR30 expression was associated with worse prognosis for BC patients treated with tamoxifen as compared to tamoxifen-naïve patients [24]. In the present study, we assessed the expression of GPR30 in 688 women with metachronous CBC. In 60 of these patients, BC2 tumors developed during ongoing tamoxifen treatment for the first BC, strongly arguing for acquired tamoxifen resistance in these tumors. We hypothesized that if GPR30 contributes to tamoxifen resistance, tumors developed under exposure of tamoxifen would exhibit higher GPR30 expression as a result of selection pressure. In the whole cohort, no difference in total GPR30 staining was observed between the tamoxifen-naïve BC2 and the presumably tamoxifen-resistant BC2. As recent studies suggest that GPR30 may have unique functions when expressed specifically in the PM [12, 20, 34, 35], we also assessed if PM-localization of GPR30 may be involved in resistance mechanisms. In a subgroup analysis, we found that BC2 diagnosed during tamoxifen treatment for BC1 was more likely to express PM-specific GPR30 than BC2 diagnosed after completed treatment. This trend was seen both in ER-positive and ER-negative cases, and could hence not only be explained by a selection of ER-negative BC2 during tamoxifen exposure (ER-negativity associated to GPR30_PM_+) [17].

To address if GPR30 expression decreases the benefit of tamoxifen, we performed a survival analysis of patients treated with tamoxifen after the diagnosis of an ER-positive BC2, stratified by GPR30 expression. In this material, GPR30 did not associate with worse prognosis. Instead, a trend was noted that among patients not treated with tamoxifen for their ER-positive BC2, PM-localized GPR30 has a higher hazard. Additionally, there was a trend that patients with PM-localized GPR30 staining in an ER-positive BC2 have a greater benefit from tamoxifen treatment. In summary, our data provides no clear evidence that tamoxifen exposure affects the prognostic value of GPR30 in BC2, or that the benefit of tamoxifen depends on GPR30 expression. However, results are not unanimous and the relationship between PM-localized GPR30 and tamoxifen needs to be further addressed in future studies.

We also explored GPR30 through disease progression. The relationship between GPR30 staining in paired BC1 and BC2 appeared to be stochastic, in line with CBC being most often considered an independent primary tumor [36]. On the other hand, LGL-metastases are seeded from, and hence clonally related to, the primary BC. GPR30 staining in the LGL was significantly lower than that observed in the corresponding BC, a pattern observed previously and suggested to reflect a successive downregulation of the receptor during cancer progression [17, 37]. However, a study on samples of normal breast tissue, invasive BC and LGL reported the mean GPR30 expression in normal breast and BC to be equal, but that the expression decreased in the LGL [37]. Unexpectedly given this background, we also show that PM-specific staining in the LGL associates with aggressive tumor characteristics and significantly higher risk of BCD, which would suggest that high GPR30 expression in the LGL is beneficial for tumor cells. Whether lower GPR30 expression is beneficial for tumor cell dissemination to the lymph nodes, or a result of environmental factors in the lymph nodes, is an interesting question for future studies.

A limited amount of data exists regarding the expression of GPR30 in healthy breast tissue. However, one study assessed GPR30 in breast tissue from 12 healthy donors in which all were defined as GPR30 positive, with strong cytoplasmic GPR30 expression in ductal and lobular epithelium, myoepithelium, and stromal fibroblasts, but no expression in smooth muscle or vascular endothelium [16]. In addition, The Human Protein Atlas database reports the level of GPR30 mRNA in normal breast tissue to be at medium level, and protein expression of GPR30 at strong levels in breast myoepithelial cells, but not detected in adipocytes or glandular cells (Data available from v19.3; www.proteinatlas.org [38]). Based on this, it seems unlikely that the mere overexpression of GPR30 could explain the pathological turn the receptor seems to undergo during BC progression. This raises the question if a transforming event changes the localization and function of GPR30, in turn yielding tumor-promoting activity in a minority of BCs. Several GPCR mutations that affect their function have been identified in cancer [39]. However, very little is still known about cancer-related mutations in GPR30. Nevertheless, it has been reported that promoter methylation suppresses GPR30 expression in both BC cell lines and primary BC tissue, and that methylation pattern of *GPER1* differ between BC tissue and healthy controls [40, 41]. Among 996 BC samples available in The Cancer Genome Atlas/PanCancer Atlas, genetic alterations in the *GPER1* gene are present in less than 2% [42–45]. Hence, it is unlikely that *GPER1* mutation causes PM-GPR30 expression, which according to data from this study and earlier is present in around 7–23% of BCs [17]. However, altered methylation may partly explain any BC-specific alterations in GPR30 expression level.

As seen here and previously [17], strong total and PM-specific GPR30 staining associate with ER-negative status. As ER-negativity is a strong marker of poor prognosis, we sought to evaluate if GPR30 adds any prognostic information beyond ER-status. The collinearity between GPR30 and ER is reflected in Cox regression adjusted for only ER (S1 Table), where the effects of both total and PM-specific GPR30 staining are reduced. However, when evaluating the association between GPR30 staining on BC outcome with the cohort stratified for ER status (S3 Fig), we found a trend that both strong total GPR30 and PM-specific GPR30 staining associate with worse prognosis in ER-positive CBC (S2 Fig), suggesting that GPR30 adds prognostic information beyond ER, at least in ER-positive tumors. Even though no prognostic effect of total or PM-specific GPR30 was seen in ER-negative CBC, tests of statistical interaction showed no significant difference in the prognostic effect of GPR30 in the ER-positive and ER-negative groups. Thus, our data suggest that although GPR30 and ER are strongly associated, GPR30 status adds prognostic information beyond ER.

Although associated with the same ligand, ER and GPR30 manifest considerable differences in terms of cellular function. ER is a nuclear receptor; a ligand-activated transcription factor that upon binding estrogen dimerizes and translocates to the nucleus, where it alters expression of target genes [46]. On the other hand, GPR30 is a G protein-coupled receptor (GPCR), and as such an integral membrane protein. In contrast to ER, an active GPCR orchestrates rapid downstream signaling by modifying the activity of several effectors and second messengers [47]. The versatile nature of a GPCR allows it to communicate with a broad signaling network, the profile of which is dependent on the expression profile of the cell. Studies have coupled GPR30 to several signaling events through both G protein-dependent and -independent mechanisms and both in response to estrogen and constitutively. Estrogen was reported to receptor-dependently stimulate increases in intracellular Ca^2+^ [13, 19], cAMP production [12], and ERK1/2 activity [12, 48–50], the latter through EGFR transactivation [12, 48], and cFos expression [49]. Furthermore, constitutively the receptor was reported to inhibit cAMP production [34] and the Ca^2+^-pump plasma membrane Ca^2+^-ATPase 4b [35], and stimulate ERK1/2 activity, the latter through PI3K [20]. Interestingly, many of these effects, including modulation of cAMP production and stimulation of ERK1/2 activity via PI3K and EGFR transactivation, have been found to depend on PM localization of the receptor. In this study, we confirm our previous results that the prognostic potential of GPR30 is more pronounced when the receptor is expressed in the PM [17]. The subcellular distribution of GPR30 is complex, with *in vitro* studies showing that receptor activity occurs both in the PM [12, 20, 34, 35], endoplasmic reticulum [13], and nucleus [51]. Today, GPR30 function is best described in the PM, which is typical for a GPCR [47], whereas few if any functions have been described in the endoplasmatic reticulum or nucleus.

Recent *in vitro* data have demonstrated that GPR30-mediated ERK1/2 signaling depends on an amino acid sequence at the receptor intracellular C-terminal end, through which the receptor interacts with scaffold and adaptor proteins localized at the PM [20], and this favors receptor PM localization [20, 34, 35, 52]. Together, these results present a model where intracellular scaffold and adaptor proteins contribute to cell proliferation by retaining GPR30 in the PM, thus spatially positioning the receptor to communicate with the ERK1/2 and EGFR pathways. As both ERK1/2 and EGFR activities are hallmarks of cell proliferation, an intriguing theory is that these interactions contribute to the association of PM-localized GPR30 with high Ki67 and poor BC outcome observed in this CBC material. Therefore, it is well motivated to further study the contribution of scaffold protein interactions to the function of GPR30 in BC pathology, and the potential of PM-localized GPR30 in targeted treatment strategies should be investigated.

In conclusion, this study evaluated the estrogen-responsive receptor GPR30 in a unique and large cohort of CBC with long-term follow-up, serving as a model for tamoxifen exposure and resistance. We conclude that GPR30 has prognostic value in CBC. On the other hand, we find no clear evidence that GPR30 is involved in tamoxifen resistance. GPR30 staining correlates with BC subtype, with the highest total and PM-specific GPR30 observed in triple-negative BC. Additionally, GPR30 is most active in CBC when located in the PM. Thus, PM-localized GPR30 is an interesting candidate for future therapeutic exploitation.

## Supporting information

S1 Raw images (PDF)

S1 Fig GPR30 antibody specificity. *A*, MCF7 cell lysates were immunoblotted with goat GPR30 antibody as previously described [20, 29, 34]. *B*, HeLa TET-On/Off cells were incubated without (-TET) or with 100 ng/ml tetracycline (+TET) for 12 h and then lysed and immunoblotted with GPR30 antibody. *C*, MCF7 cells were stained live with GPR30 antibody for 30 min and then fixed and stained with Alexa488-labeled anti-goat antibodies (Life Technologies) as previously described [20, 29, 34]. *D*, MCF7 cells were transiently transfected with a plasmid containing the cDNA of human GPR30 tagged in the N-terminus with the FLAG tag (FLAG-hGPR30), stained live with mouse M1 FLAG antibodies (Sigma-Aldrich) for 30 min, and then fixed and stained with Alexa488-labeled mouse IgG2b antibodies (Life Technologies) as previously described [29, 34]. In *C* and *D*, 4',6-Diamidino-2-phenylindole (DAPI) was used for nuclear staining, and fluorescence images were collected using a Nikon Eclipse confocal microscope. The results are representative of experiments performed at least three times. *Bar*, 10 μm.
(EPS)

S2 Fig Cumulative incidence of BCD in CBC patients in relation to GPR30 staining of BC2, stratified by ER status of BC2. Cumulative incidence of competing event (death from other cause than BC) is shown for comparison. HR values were estimated using a cause-specific Cox proportional hazards model, and values of *p* were calculated using Wald test. *A*-*B*, cumulative incidence of BCD in relation to GPR30_TOT_ in CBC patients with ER-positive BC2 (*A*) or ER-negative BC2 (*B*). *C*-*D*, cumulative incidence of BCD in relation to GPR30_PM_ in CBC patients with ER-positive BC2 (*C*) or ER-negative BC2 (*D*).
(PDF)

S3 Fig Cumulative incidence of BCD in relation to GPR30 staining of the tumor pair (BC1/BC2). Presented HR values were estimated using Cox proportional hazards model and p values were calculated using Wald test, where the groups with weak/weak GPR30_TOT_ and PM-/PM- GPR30_PM_ status were used as reference groups. *A*, cumulative incidence of BCD in relation to GPR30_TOT_ in the tumor pair. *B*, cumulative incidence of BCD in relation to GPR30_PM_ in the tumor pair.
(PDF)

S4 Fig Cumulative incidence of BCD in CBC patients with ER+ BC2 in relation to tamoxifen treatment of BC2, stratified by GPR30 expression of BC2. Cumulative incidence of competing event (death from other cause than BC) is shown for comparison. HR values were estimated using a cause-specific Cox proportional hazards model, and values of *p* were calculated using Wald test. *A*-*D*, cumulative incidence of BCD in relation to tamoxifen treatment of BC2 in CBC patients with weak GPR30 staining (*A*) or strong GPR30 staining (*B*), and in patients without PM-specific GPR30 staining (*C*) and in patients with PM-specific staining (GPR30_PM_+) (*D*).
(PDF)

S1 Table Forest plots presenting estimates of the prognostic impact of GPR30 in multivariate analyses adjusted for only one variable per row. *A*, multivariate analyses of strong total GPR30 (GPR30_TOT_) adjusted for each variable separately. *B*, multivariate analyses of plasma membrane-specific GPR30 (GPR30_PM_+) adjusted for each variable separately.
(PDF)

S2 Table Relationship between total GPR30 staining (GPR30_TOT_) and PM-specific GPR30 (GPR30_PM_) staining of BC2 and risk of death from BC after CBC diagnosis in patients stratified by ER status and tamoxifen treatment, and interactions between GPR30 and ER or tamoxifen. Prognostic effect was calculated by cox proportional hazards model with Wald test. Interaction between GPR30 and the stratifying variable (ER or tamoxifen) was assessed using an interaction test. Relationship between GPR30 and risk of death from BC were assessed by Cox regressions.
(PDF)

S3 Table Relationship between GPR30 staining of the tumor pair (BC1/BC2) and risk of death from BC after CBC diagnosis. Prognostic effect was calculated by cox proportional hazards model with Wald test. The groups with weak/weak GPR30_TOT_ and PM-/PM- GPR30_PM_ status were used as reference groups for survival analyses. Relationship between GPR30 and risk of death from BC were assessed by Cox regressions.
(PDF)
