# Supplementary material for: Plasma membrane expression of G protein-coupled estrogen receptor (GPER)/G protein-coupled receptor 30 (GPR30) is associated with worse outcome in metachronous contralateral breast cancer
Source: PLoS One. 2020 Apr 17;15(4):e0231786. doi: 10.1371/journal.pone.0231786 (PMC7164601; doi:10.1371/journal.pone.0231786)
Supplement: S1 Raw images — (PDF) [file pone.0231786.s001.pdf]

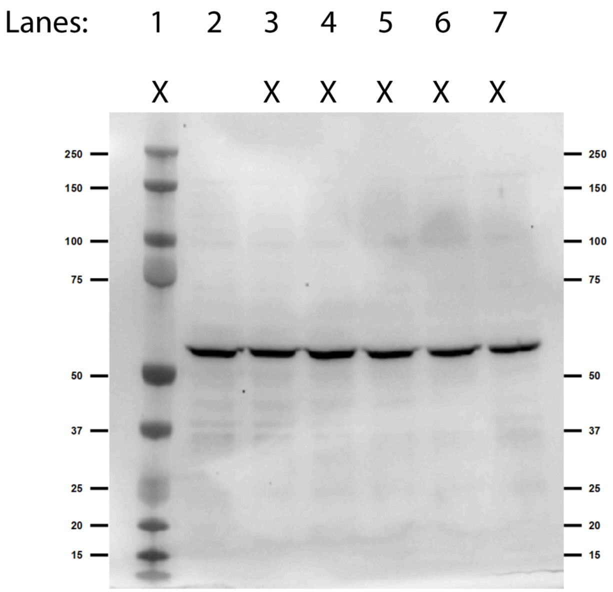

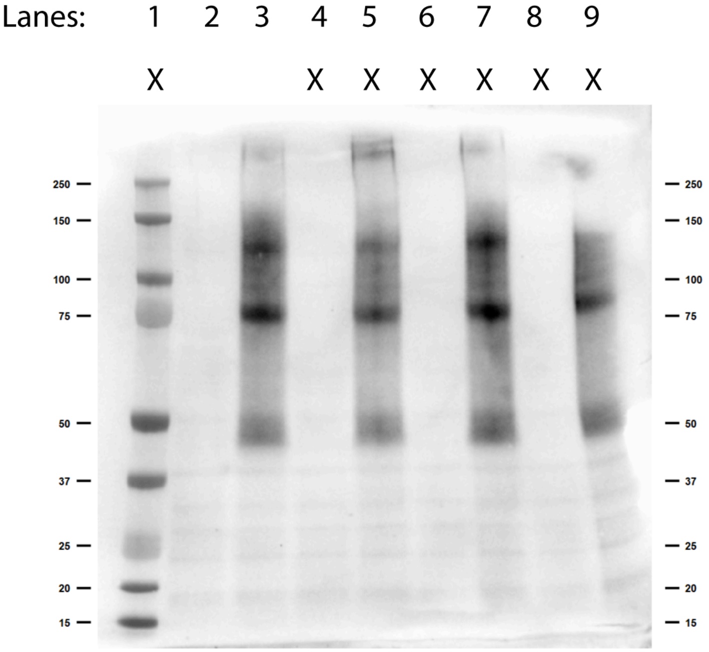

Figure panel: S1_Fig, panel A

Lanes:
Lane 1, molecular mass markers
Lanes 2-7, lysates of MCF7 cells

Capture method: BIO-RAD ChemiDoc™ MP Imaging System

Figure panel: S1_Fig, panel B

Lanes:
Lane 1, molecular mass markers
Lanes 2,4,6,8, lysates of HeLa Tet-ON/OFF cells not treated with Tet
Lanes 3,5,7,9, lysates of HeLa Tet-ON/OFF cells treated with Tet

Capture method: BIO-RAD ChemiDoc™ MP Imaging System
