# Supplementary material for: Plasma membrane expression of G protein-coupled estrogen receptor (GPER)/G protein-coupled receptor 30 (GPR30) is associated with worse outcome in metachronous contralateral breast cancer
Source: PLoS One. 2020 Apr 17;15(4):e0231786. doi: 10.1371/journal.pone.0231786 (PMC7164601; doi:10.1371/journal.pone.0231786)
Supplement: S2 Fig — Cumulative incidence of competing event (death from other cause than BC) is shown for comparison. HR values were estimated using a cause-specific Cox proportional hazards model, and values of p were calculated using Wald test. A-B, cumulative incidence of BCD in relation to GPR30TOT in CBC patients with ER-positive BC2 (A) or ER-negative BC2 (B). C-D, cumulative incidence of BCD in relation to GPR30PM in CBC patients with ER-positive BC2 (C) or ER-negative BC2 (D). (PDF) [file pone.0231786.s003.pdf]

A) Total GPR30 staining of ER−positive BC2

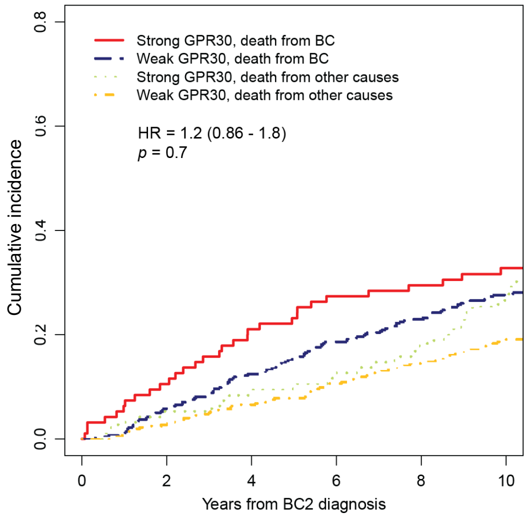

Number at risk

**Strong GPR30**	95	80	67	57	50	34
**Weak GPR30**	397	363	322	281	246	194

B) Total GPR30 staining of ER−negative BC2

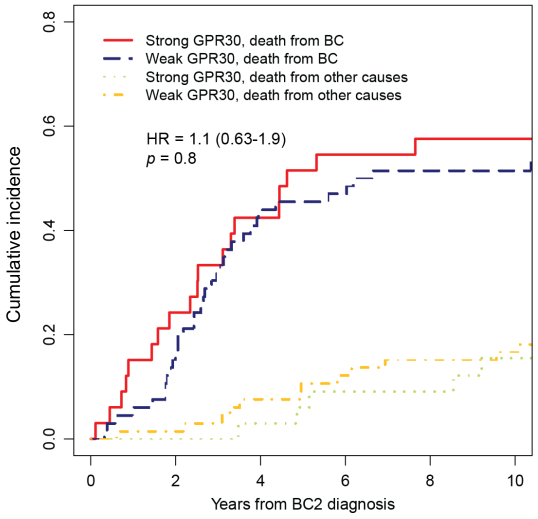

Number at risk

**Strong GPR30**	33	25	18	12	11	8
**Weak GPR30**	66	54	32	27	22	21

C) PM−specific GPR30 staining of ER−positive BC2

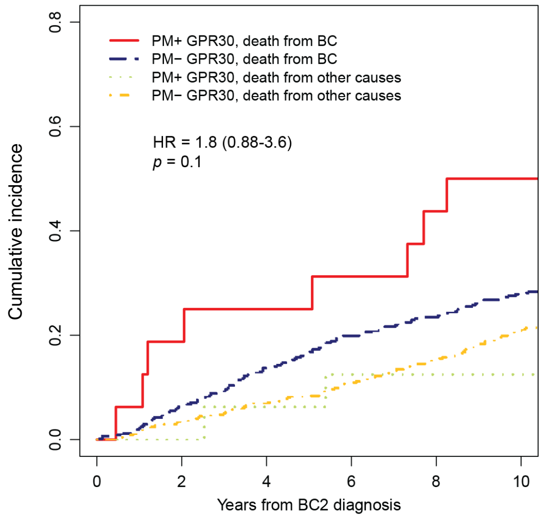

Number at risk

**PM+ GPR30**	16	13	11	9	7	6
**PM- GPR30**	476	430	378	329	289	222

D) Total GPR30 staining of ER−negative BC2

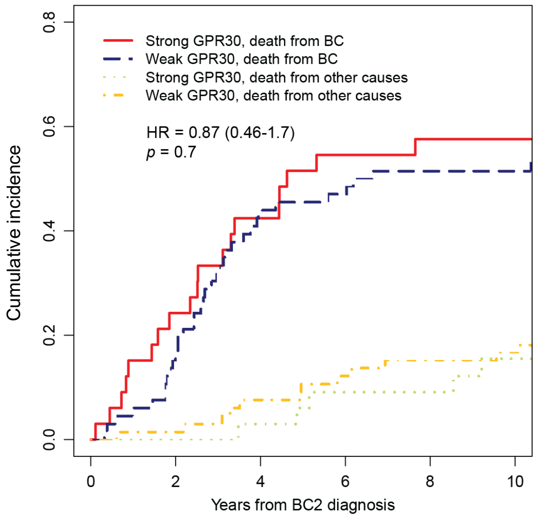

Number at risk

**PM+ GPR30**	33	25	18	12	11	8
**PM- GPR30**	66	54	32	27	22	21
