# Supplementary material for: Plasma membrane expression of G protein-coupled estrogen receptor (GPER)/G protein-coupled receptor 30 (GPR30) is associated with worse outcome in metachronous contralateral breast cancer
Source: PLoS One. 2020 Apr 17;15(4):e0231786. doi: 10.1371/journal.pone.0231786 (PMC7164601; doi:10.1371/journal.pone.0231786)
Supplement: S3 Fig — Presented HR values were estimated using Cox proportional hazards model and p values were calculated using Wald test, where the groups with weak/weak GPR30TOT and PM-/PM- GPR30PM status were used as reference groups. A, cumulative incidence of BCD in relation to GPR30TOT in the tumor pair. B, cumulative incidence of BCD in relation to GPR30PM in the tumor pair. (PDF) [file pone.0231786.s004.pdf]

A) **GPR30 intensity in the CBC pair (BC1/BC2)**

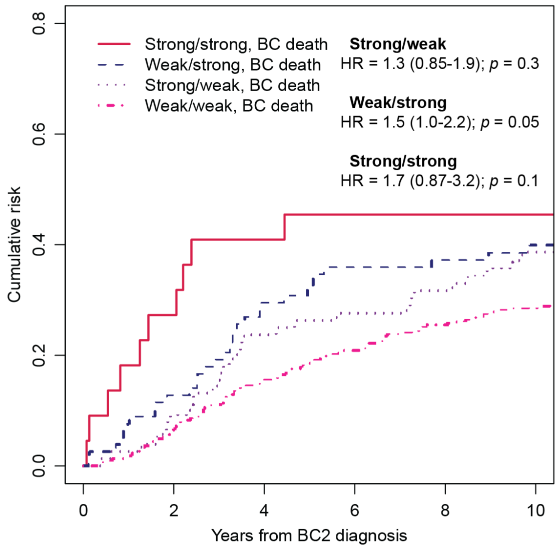

Number at risk

**Weak/weak**	302	274	232	207	178	145
**Weak/strong**	78	63	47	39	26	24
**Strong/weak**	76	67	54	45	39	30
**Strong/strong**	22	16	13	11	9	8

B) **PM−specific GPR30 in the CBC pair (BC1/BC2)**

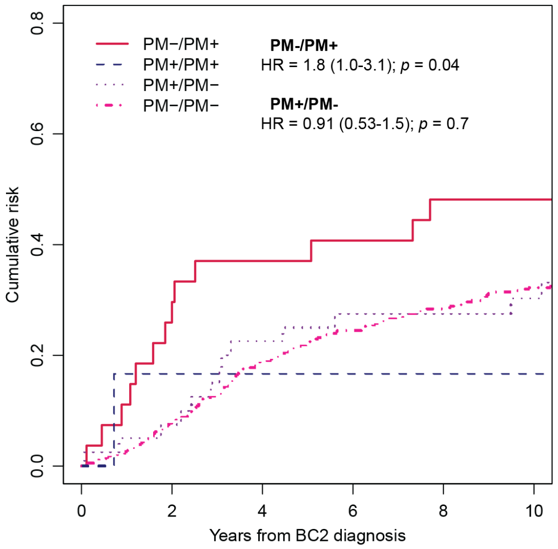

Number at risk

**PM-/PM-**	405	360	298	260	228	177
**PM-/PM+**	27	19	14	12	10	10
**PM+/PM-**	40	37	31	27	21	17
**PM+/PM+**	6	4	3	3	3	3
