# Supplementary material for: Plasma membrane expression of G protein-coupled estrogen receptor (GPER)/G protein-coupled receptor 30 (GPR30) is associated with worse outcome in metachronous contralateral breast cancer
Source: PLoS One. 2020 Apr 17;15(4):e0231786. doi: 10.1371/journal.pone.0231786 (PMC7164601; doi:10.1371/journal.pone.0231786)
Supplement: S4 Fig — Cumulative incidence of competing event (death from other cause than BC) is shown for comparison. HR values were estimated using a cause-specific Cox proportional hazards model, and values of p were calculated using Wald test. A-D, cumulative incidence of BCD in relation to tamoxifen treatment of BC2 in CBC patients with weak GPR30 staining (A) or strong GPR30 staining (B), and in patients without PM-specific GPR30 staining (C) and in patients with PM-specific staining (GPR30PM+) (D). (PDF) [file pone.0231786.s005.pdf]

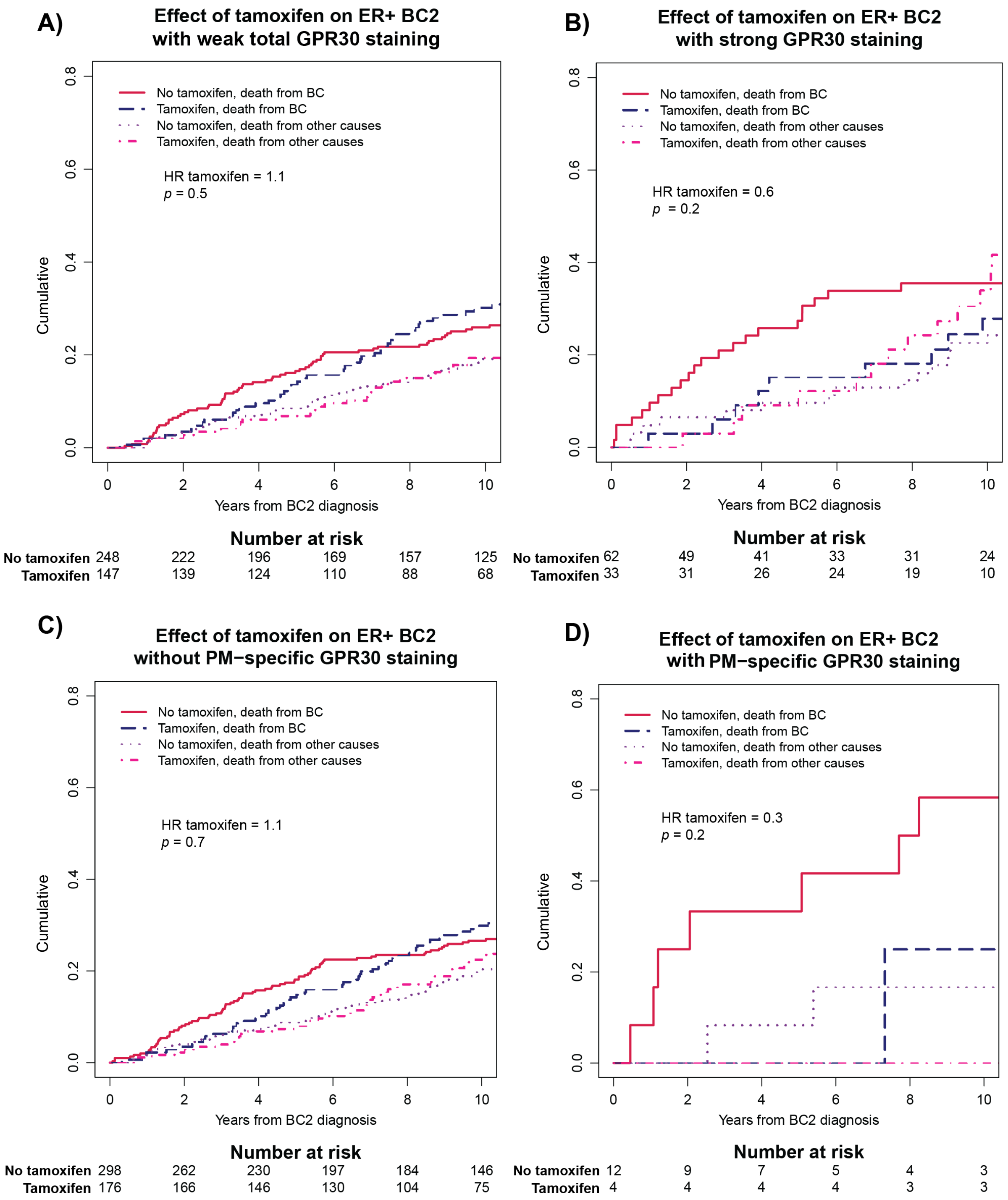
A)
Effect of tamoxifen on ER+ BC2
with weak total GPR30 staining
No tamoxifen, death from BC
Tamoxifen, death from BC
No tamoxifen, death from other causes
Tamoxifen, death from other causes
HR tamoxifen = 1.1
p = 0.5
Cumulative
0.0 0.2 0.4 0.6 0.8
0 2 4 6 8 10
Years from BC2 diagnosis
Number at risk
No tamoxifen 248 222 196 169 157 125
Tamoxifen 147 139 124 110 88 68
B)
Effect of tamoxifen on ER+ BC2
with strong GPR30 staining
No tamoxifen, death from BC
Tamoxifen, death from BC
No tamoxifen, death from other causes
Tamoxifen, death from other causes
HR tamoxifen = 0.6
p = 0.2
Cumulative
0.0 0.2 0.4 0.6 0.8
0 2 4 6 8 10
Years from BC2 diagnosis
Number at risk
No tamoxifen 62 49 41 33 31 24
Tamoxifen 33 31 26 24 19 10
C)
Effect of tamoxifen on ER+ BC2
without PM−specific GPR30 staining
No tamoxifen, death from BC
Tamoxifen, death from BC
No tamoxifen, death from other causes
Tamoxifen, death from other causes
HR tamoxifen = 1.1
p = 0.7
Cumulative
0.0 0.2 0.4 0.6 0.8
0 2 4 6 8 10
Years from BC2 diagnosis
Number at risk
No tamoxifen 298 262 230 197 184 146
Tamoxifen 176 166 146 130 104 75
D)
Effect of tamoxifen on ER+ BC2
with PM−specific GPR30 staining
No tamoxifen, death from BC
Tamoxifen, death from BC
No tamoxifen, death from other causes
Tamoxifen, death from other causes
HR tamoxifen = 0.3
p = 0.2
Cumulative
0.0 0.2 0.4 0.6 0.8
0 2 4 6 8 10
Years from BC2 diagnosis
Number at risk
No tamoxifen 12 9 7 5 4 3
Tamoxifen 4 4 4 4 3 3
