# Supplementary material for: Plasma membrane expression of G protein-coupled estrogen receptor (GPER)/G protein-coupled receptor 30 (GPR30) is associated with worse outcome in metachronous contralateral breast cancer
Source: PLoS One. 2020 Apr 17;15(4):e0231786. doi: 10.1371/journal.pone.0231786 (PMC7164601; doi:10.1371/journal.pone.0231786)

A) **Hazard ratio of strong total GPR30 staining in BC2**

Adjustment	HR	n	Events	95% CI	p
GPR30TOT staining of BC1	1.44	478	189	1.0-2.0	0.03
Size of BC1	1.31	539	210	0.95-1.8	0.1
LGL status of BC1	1.34	542	218	0.98-1.8	0.07
HER2 status of BC1	1.43	499	195	1.0-2.0	0.03
Ki67 intensity of BC1	1.42	498	194	1.0-2.0	0.04
ER status of BC1	1.35	507	196	0.97-1.9	0.07
Tumor size of BC2	1.53	582	230	1.1-2.1	0.006
LGL status of BC2	1.38	495	207	1.0-1.9	0.05
HER2 status of BC2	1.33	589	236	0.98-1.8	0.06
Ki67 intensity of BC2	1.40	589	236	1.0-1.9	0.03
Age at CBC diagnosis	1.34	595	237	1.0-1.8	0.05
Year of CBC diagnosis	1.33	595	237	0.99-1.8	0.06
Interval between BC1 and BC2	1.34	595	237	0.99-1.8	0.06
ER status of BC2	1.18	595	237	0.87-1.6	0.3
**All the above (exc. ER BC2)**	**1.46**	**350**	**145**	**0.98-2.2**	**0.06**

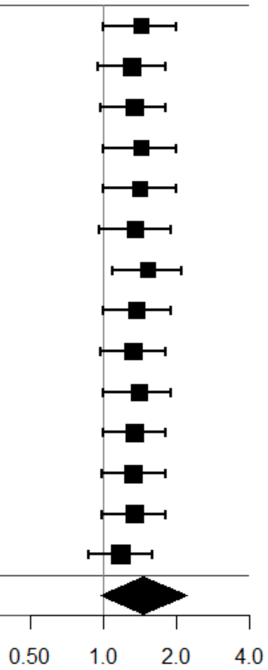

B) **Hazard ratio of PM+ GPR30 staining in BC2**

Adjustment	HR	n	Events	95% CI	p
GPR30TOT staining of BC1	1.70	478	189	1.0-2.8	0.04
Size of BC1	1.85	539	210	1.1-3.1	0.02
LGL status of BC1	1.80	542	218	1.1-2.9	0.022
HER2 status of BC1	1.84	499	195	1.1-3.0	0.02
Ki67 intensity of BC1	1.58	498	194	0.94-2.6	0.08
ER status BC1	1.63	507	196	0.96-2.7	0.07
Tumor size of BC2	1.87	582	230	1.2-3.0	0.009
LGL status of BC2	2.08	495	206	1.3-3.4	0.003
HER2 status of BC2	1.64	589	236	1.0-2.6	0.04
Ki67 intensity of BC2	1.29	583	234	0.80-2.1	0.3
Age at CBC diagnosis	1.69	595	237	1.1-2.7	0.03
Interval of CBC diagnosis	1.65	595	237	1.0-2.6	0.04
Interval between BC1 and BC2	1.70	595	237	1.1-2.7	0.03
ER status of BC2	1.10	591	237	0.65-1.8	0.8
**All the above (exc. ER BC2)**	**1.90**	**350**	**145**	**1.0-3.4**	**0.04**

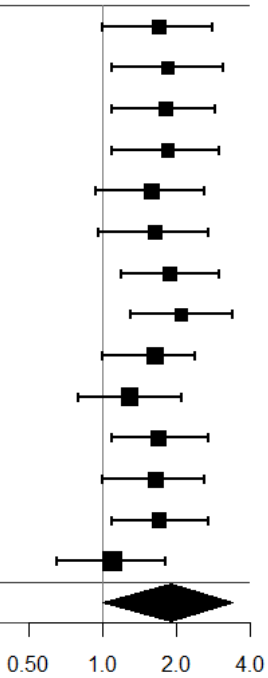

Supplement: S1 Table — A, multivariate analyses of strong total GPR30 (GPR30TOT) adjusted for each variable separately. B, multivariate analyses of plasma membrane-specific GPR30 (GPR30PM+) adjusted for each variable separately. (PDF) [file pone.0231786.s006.pdf]
