# Supplementary material for: Plasma membrane expression of G protein-coupled estrogen receptor (GPER)/G protein-coupled receptor 30 (GPR30) is associated with worse outcome in metachronous contralateral breast cancer
Source: PLoS One. 2020 Apr 17;15(4):e0231786. doi: 10.1371/journal.pone.0231786 (PMC7164601; doi:10.1371/journal.pone.0231786)
Supplement: S2 Table — Prognostic effect was calculated by cox proportional hazards model with Wald test. Interaction between GPR30 and the stratifying variable (ER or tamoxifen) was assessed using an interaction test. Relationship between GPR30 and risk of death from BC were assessed by Cox regressions. (PDF) [file pone.0231786.s007.pdf]

		Unadjusted						Adjusted for BC1[1]					Adjusted for BC1 and BC2[2]				
	**Patient group**	**HR**	**n**	**Events**	**95% CI**	p_{HR}	p_i	**HR**	**n**	**Events**	**95% CI**	p_{HR}	**HR**	**n**	**Events**	**95% CI**	p_{HR}
**$GPR30_{TOT}$**	ER+ BC2	1.2	492	177	0.86-1.8	0.2	0.7	0.95	349	126	0.59-1.5	0.8	1.1	282	105	0.66-1.9	0.7
	ER- BC2	1.1	99	60	0.63-1.9	0.8		1.4	75	45	0.74-2.7	0.3	3.0	67	40	1.4-6.6	0.006
	ER+, tamoxifen for BC2	1.4	70	35	0.60-3.5	0.4	0.4	0.55	123	45	0.18-1.6	0.3	1.1	45	24	0.30-4.0	0.26
	ER+, no tamoxifen for BC2	1.3	48	26	0.39-4.4	0.7		1.1	224	79	0.94-1.9	0.8	7.9	35	21	1.1-60	0.04
**$GPR30_{PM}$**	ER+ BC2	1.8	492	177	0.88-3.6	0.1	0.1	1.8	349	126	0.80-3.9	0.2	1.9	282	105	0.76-4.5	0.2
	ER- BC2	0.87	99	60	0.45-1.7	0.7		1.1	75	45	0.47-2.4	0.8	1.3	67	40	0.55-3.2	0.5
	ER+, tamoxifen for BC2	0.51	180	63	0.07-3.7	0.5	0.3	0.27	123	45	0.030-2.5	0.3	0.57	104	40	0.06-5.3	0.6
	ER+, no tamoxifen for BC2	2.8	310	112	1.3-6.0	0.01		2.2	224	79	0.90-5.6	0.08	3.8	176	63	1.4-11	0.01

[1] Adjusted for GPR30 intensity, age and calendar interval of diagnosis, tumor size, node status, HER2 status and Ki67 staining of BC1. [2] Adjusted for GPR30 intensity, size, node status, HER2 status and ki67 staining of BC1 and BC2, as well as age and calendar interval at BC2 diagnosis and interval between BC1 and BC2.

p_i: $p_{interaction}$. Level of evidence that GPR30 has different effect in the compared groups.
