# Supplementary material for: Plasma membrane expression of G protein-coupled estrogen receptor (GPER)/G protein-coupled receptor 30 (GPR30) is associated with worse outcome in metachronous contralateral breast cancer
Source: PLoS One. 2020 Apr 17;15(4):e0231786. doi: 10.1371/journal.pone.0231786 (PMC7164601; doi:10.1371/journal.pone.0231786)
Supplement: S3 Table — Prognostic effect was calculated by cox proportional hazards model with Wald test. The groups with weak/weak GPR30TOT and PM-/PM- GPR30PM status were used as reference groups for survival analyses. Relationship between GPR30 and risk of death from BC were assessed by Cox regressions. (PDF) [file pone.0231786.s008.pdf]

	Patient group	Unadjusted					Adjusted for BC1[1]					Adjusted for BC1 and BC2[2]				
	**Patient group**	**HR**	**n**	**Events**	**95% CI**	***p***	**HR**	**n**	**Events**	**95% CI**	***p***	**HR**	**n**	**Events**	**95% CI**	***p***
**$GPR30_{TOT}$**	Strong/weak	1.3	378	145	0.85-1.9	0.3	1.6	337	132	1.0-2.5	0.03	1.4	274	110	0.87-2.24	0.2
	Weak/strong	1.5	380	147	1.0-2.2	0.05	1.2	343	133	0.80-1.9	0.3	1.4	278	112	0.89-2.3	0.1
	Strong/strong	1.7	324	123	0.87-3.2	0.1	1.9	292	112	0.91-3.8	0.09	1.8	235	91	0.78-4.1	0.1
**$GPR30_{PM}$**	PM+/PM-	0.91	445	173	0.53-1.5	0.7	0.78	397	156	0.43-1.4	0.4	0.90	323	131	0.45-1.8	0.8
	PM-/PM+	1.8	432	172	1.0-3.1	0.04	2.1	387	155	1.1-3.8	0.02	1.85	317	131	0.96-3.6	0.07

[1] Adjusted for age and calendar interval of diagnosis, tumor size, node status, ER status, HER2 status and Ki67 staining of BC1. Analyses of $GPR30_{PM}$ were also adjusted for GPR30 intensity of BC1. [2] Adjusted for size, node status, ER status, HER2 status and ki67 staining of BC1 and BC2, as well as age and calendar interval at BC2 diagnosis and interval between BC1 and BC2. Analyses of $GPR30_{PM}$ were also adjusted for GPR30 intensity of BC2.
